# Supplementary material for: Multiple ETS Factors Participate in the Transcriptional Control of TERT Mutant Promoter in Thyroid Cancers
Source: Cancers (Basel). 2022 Jan 12;14(2):357. doi: 10.3390/cancers14020357 (PMC8774187; doi:10.3390/cancers14020357)
Supplement: Supplementary file 1 [file cancers-14-00357-s001.zip › Thornton et al. Supplementary Tables.pdf]

Supplementary Tables.

Supplementary Table S1. List of short hairpin RNA (shRNA) clones.

Clone ID (from GPP Web Portal)	Clone category	Target Sequence	Vector backbone	Original Target Gene ID	Original Target Gene Symbol	Match Regions
TRCN0000017078	TRC 1.0	CCGAGCTATGAGATATTACTA	pLKO.1	26298	EHF	CDS
TRCN0000013856	TRC 1.0	CCTGCCGTAATTGTGGAACAT	pLKO.1	1997	ELF1	5UTR, CDS
TRCN0000273855	TRC 2.0	GTCTCCTGAGTTAGGTATAAA	pLKO_TRC005	1998	ELF2	CDS
TRCN0000013863	TRC 1.0	CTCCCTAATTTATGTGCTATA	pLKO.1	1999	ELF3	3UTR
TRCN0000013869	TRC 1.0	CGCGGAAGTCTTACTCAATAT	pLKO.1	2000	ELF4	CDS
TRCN0000007450	TRC 1.0	CCCAAGAGTAACTCTCATTAT	pLKO.1	2002	ELK1	3UTR
TRCN0000329766	TRC 2.0	ACTCGTCCTTCACCATTAATT	pLKO_TRC005	2004	ELK3	CDS
TRCN0000013886	TRC 1.0	GCTTCTCTTACACCAGCATTT	pLKO.1	2005	ELK4	3UTR, CDS
TRCN0000013909	TRC 1.0	CCTGCGCTATTACTATAACAA	pLKO.1	2077	ERF	CDS
TRCN0000013914	TRC 1.0	GCTCATATCAAGGAAGCCTTA	pLKO.1	2078	ERG	CDS
TRCN0000005591	TRC 1.0	CTGGAATTACTCACTGATAAA	pLKO.1	2113	ETS1	CDS
TRCN0000233984	TRC 2.0	GAGCAAGGCAAACCAGTTATT	pLKO_TRC005	23872	Ets2	CDS
TRCN0000013918	TRC 1.0	GCCGACTAAGAGAAGTTGTAA	pLKO.1	2114	ETS2	3UTR
TRCN0000013923	TRC 1.0	GTGGGAGTAATCTAAACATTT	pLKO.1	2115	ETV1	3UTR
TRCN0000013928	TRC 1.0	CCTCTTGACTTTCCTGGTTAT	pLKO.1	2117	ETV3	3UTR
TRCN0000013934	TRC 1.0	GCAGAGCTTTAAGCAAGAATA	pLKO.1	2118	ETV4	CDS
TRCN0000273931	TRC 2.0	GAGCGATACGTCTACAAATTT	pLKO_TRC005	2119	ETV5	CDS
TRCN0000003852	TRC 1.0	AGGAGCTGGATGAACAAATAT	pLKO.1	2120	ETV6	CDS
TRCN0000016743	TRC 1.0	CCAGATGTGAAGCTCAAATTA	pLKO.1	51513	ETV7	5UTR, CDS
TRCN0000005323	TRC 1.0	CCCATGAACTACAACAGCTAT	pLKO.1	2313	FLI1	CDS
TRCN0000304469	TRC 2.0	AGCTTAGTGTACAGGTAATTT	pLKO_TRC005	14390	Gabpa	CDS
TRCN0000304508	TRC 2.0	ATGAACCAATAGGCAATTTAA	pLKO_TRC005	14390	Gabpa	CDS

Abbreviations: GPP= Genetic Perturbation Platform; CDS= coding DNA sequence; UTR= untranslated region

Supplementary Table S2. List of primers used for quantitative PCR.

**qPCR Primer List 5' -> 3'**		
**Target Gene**	**Forward**	**Reverse**
ETS1	TACACAGGCAGTGGACCATC	CCCCGCTGTCCTGTGGATG
ETS2	CCCCTGTGGCTAACAGTTACA	AGGTAGCTTTTAAGGCTTGACTC
ELF3	CAACTATGGGGCCAAAAGAA	CAACCCTCAGTTCCGACTCT
ELF1	TGTCCAACAGAACGACCTAGT	GGCAGGAAAAATAGCTGGATCAC
ELF2	AAACTGTAGTGGAGGTGTCAACT	CATGGCTATCTGGTGATGTTGG
ETV7	TCAGCTGCTCCTTGATACCC	GCCCCGGTTCCTTCTTAAT
ELF4	CCTGATCTTTGAGTTCGCAAGC	AGTCCCGAGTACAGATGCAGT
ETV1	GGCCCCAGGCAGTTTTATGAT	GATCCTCGCCGTTGGTATGT
ETV3	GGTGGAGGGTATCAGTTTCCT	TGATGAATGGGTAGTTGGGCAT
ETV4	CAGTGCCTTTACTCCAGTGCC	CTCAGGAAATTCCGTTGCTCT
ELK1	TCCCTGCTTCCTACGCATACA	GCTGCCACTGGATGGAAACT
ETV5	CAGTCAACTTCAAGAGGCTTGG	TGCTCATGGCTACAAGACGAC
ETV6	CCCATTGGGAGAATAGCAGA	CAGGGCTCTGGACATTTTCT
ERF	ATTCATTGATGTGGGGTTGG	AGATGAAGAGCAGGCTGGTG
EHF	CCACCAGTCACCTTCCTGTT	GAGCCACTGCCTCTGATTTC
ELK3	ATCTGCTGGACCTCGAACGA	TTCTGCCCGATCACCTTCTTG
ELK4	ACTCAGCCGAGCCCTCAG	GGTGGCTTTTTGGAAGGTG
GABPA	AAGAACGCCTTGGGATACCCT	GTGAGGTCTATATCGGTCATGCT
ERG	TGGCTCAAGGAACTCTCCTG	ATAACTCTGCGCTCGTTCGT
FLI1	ATGACCACCAACGAGAGGAG	GTTCCTTGCCATCCATGTTC
ACTB	CTCTTCCAGCCTTCCTTCCT	AGCACTGTGTTGGCGTACAG
